# Development of Arginine Stearate-Based Solid Lipid Nanoparticles for Enhanced Idebenone Delivery: In Vitro Evaluation on Glioblastoma Model

**DOI:** 10.3390/molecules31152642

**Published:** 2026-07-29

**Authors:** Stefania Petralito, Federica Curcio, Laura Di Muzio, Roberta Sole, Francesca Giordano, Adele Elisabetta Leonetti, Sonia Trombino, Roberta Cassano

**Affiliations:** 1 Department of Chemistry and Pharmaceutical Technologies, University of Rome “La Sapienza”, Piazzale Aldo Moro, 5, 00185 Rome, Italy; stefania.petralito@uniroma1.it (S.P.); laura.dimuzio@uniroma1.it (L.D.M.); 2 Department of Pharmacy, Health and Nutritional Science, University of Calabria, 87036 Rende, Italy; federica.curcio@unical.it (F.C.); roberta.sole@unical.it (R.S.); francesca.giordano@unical.it (F.G.);

**Keywords:** glioblastoma, solid lipid nanoparticles, idebenone, targeted drug delivery

## Abstract

Glioblastoma (GBM) is the most aggressive primary brain tumor and remains difficult to treat due to its invasive nature, therapeutic resistance, and the presence of the blood–brain barrier (BBB), which represents a major obstacle to effective drug delivery. This study describes the development of biocompatible solid lipid nanoparticles (SLNs) based on a novel arginine stearate derivative for the encapsulation of idebenone, a synthetic antioxidant with potential biological activity. The objective of this work was to design and characterize a lipid-based nanoparticulate system for idebenone delivery and to evaluate its physicochemical properties and preliminary in vitro biological effects. The nanoparticles were characterized by Dynamic Light Scattering (DLS) and Differential Scanning Calorimetry (DSC), and in vitro release profiles were investigated under different pH conditions. Antioxidant activity and cell viability assays were also performed in glioblastoma and non-tumorigenic cell lines. The results indicate successful formulation of idebenone-loaded SLNs with good encapsulation efficiency, maintained antioxidant activity, and promising physical stability over time as monitored by size analysis. The rationale behind the development of arginine stearate-based SLNSs is to optimize the performance of pharmaceutical active ingredients, such as idebenone, versus non-tumorigenic cells. Overall, these findings support the potential of the developed SLNs as a promising delivery system for further in vitro and in vivo investigations.

## 1. Introduction

Glioblastoma (GBM) is the most common and aggressive primary malignant brain tumor, classified as a grade IV astrocytoma by the World Health Organization (WHO) [1]. Originating from glial cells, which provide support to neurons, GBM is characterized by a highly infiltrative nature, rapid proliferation, and significant resistance to conventional therapies [2]. The precise etiology of this disease remains largely unknown, but is believed to be due to a complex interplay of genetic, epigenetic, and environmental factors [3]. The poor prognosis associated with GBM, with a median survival of approximately 15–20 months despite aggressive treatment, underscores the urgent need for innovative and more effective therapeutic strategies [4,5]. Patients with GBM often present with a variety of neurological symptoms, which are highly dependent on the tumor’s size and location in the brain. The most common manifestations include persistent headaches, nausea and vomiting, seizures, cognitive deficits (e.g., memory problems, language difficulties), personality changes, and focal neurological deficits such as muscle weakness [6]. The insidious onset and progressive worsening of these symptoms often lead to significant patient morbidity and severe impairment in quality of life.

Current treatment for newly diagnosed GBM typically involves maximal surgical resection followed by concurrent radiotherapy and chemotherapy with temozolomide (TMZ), followed by adjuvant TMZ [7]. Despite these intensive multimodal treatments, the intrinsic biological aggressiveness of GBM, combined with challenges such as the blood–brain barrier (BBB), a formidable physiological barrier that limits the passage of most therapeutic agents into the brain, and intrinsic or acquired drug resistance, significantly limits treatment efficacy and contributes to high relapse rates [8]. The ineffectiveness of current therapeutic strategies in substantially improving patient outcomes and life expectancy has prompted research to focus on identifying innovative therapeutic molecules and delivery methods with greater efficacy and reduced systemic side effects.

In this context, nanoparticle (NP) systems represent a highly promising strategy to overcome the limitations of conventional GBM therapies. Nanoparticles offer several advantages, including their nanoscale size, which facilitates drug delivery across biological barriers, and the possibility of surface functionalization with specific ligands to enable targeted delivery [9]. Crucially, nanoparticles (NPs) can be engineered to cross the blood–brain barrier (BBB), a formidable physiological barrier that limits the passage of most therapeutic agents into the brain, and to selectively accumulate in tumor tissue via enhanced permeability and retention (EPR) or active targeting mechanisms [10,11]. This targeted delivery can lead to controlled and sustained release of therapeutic agents directly to the tumor site, thus maximizing their therapeutic index and minimizing off-target toxicity.

This work aims to develop and characterize a novel arginine stearate-based SLN formulation for idebenone delivery and to perform a preliminary in vitro evaluation of its biological activity against glioblastoma cells. Specifically, the initial phase of this experimental work involved the design, synthesis, and comprehensive characterization of a novel functional derivative, arginine stearate. This derivative is intended for the fabrication of solid lipid nanoparticles (SLNSs), a class of lipid nanoparticles known for their biocompatibility, biodegradability, and stability [12,13]. The rationale behind the development of arginine stearate-based SLNSs is to optimize the performance of pharmaceutical active ingredients, such as idebenone. Idebenone is a strongly lipophilic (hydrophobic) molecule. Structurally, it is a synthetic analog of Coenzyme Q10 (CoQ10), but it features a shorter side chain (a hydroxydecyl group instead of the long isoprenoid chain of CoQ10). This drug is a potent antioxidant, recognized for its ability to mitigate oxidative stress by interrupting free radical chain reactions [14]. Idebenone is used in clinical settings, primarily for the treatment of rare neurodegenerative and mitochondrial diseases [15]. Its main clinical applications include: (i) Leber’s Hereditary Optic Neuropathy (LHON) [16]. This is the primary indication for which idebenone (marketed under the brand name Raxone) obtained approval from the European Medicines Agency (EMA). It is used to treat visual impairment in adolescents and adults affected by this genetic mitochondrial disease [17]; (ii) Friedreich’s Ataxia: Historically, it has been used (and is prescribed off-label in some countries) to reduce cardiac hypertrophy and improve neurological parameters in patients with this condition, although clinical trial results have shown mixed efficacy [18].

The scientific literature on idebenone is extensive; in fact, following oral administration, idebenone undergoes extensive first-pass metabolism and has a very low bioavailability of only around 1%. The use of an alternative route of administration, such as the nasal route, and its incorporation into a new carrier (nanocomposite microspheres) could resolve the problems associated with reduced absorption, stability and rapid biotransformation, thereby increasing the chances of idebenone realising its therapeutic potential. In this regard, Boyuklieva et al. have developed bioadhesive nanocomposite microparticles measuring 7.37 ± 2.4 µm, with a high mucoadhesive capacity that makes them suitable for nasal administration in the treatment of Alzheimer’s disease [19].

**Figure 1:**
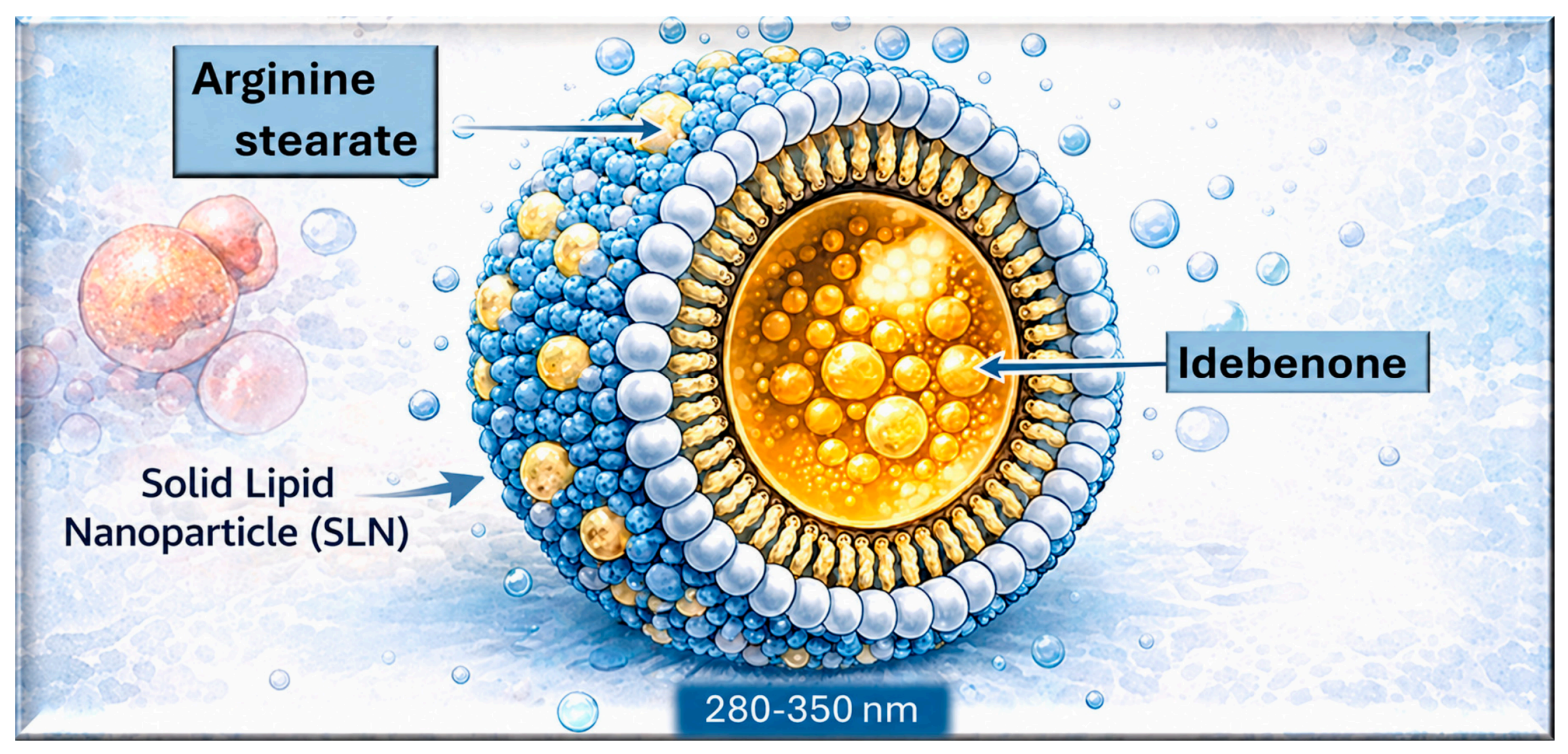
Schematic representation of arginine stearate-based SLNs containing idebenone.

By encapsulating idebenone within these novel SLNs, this research aims to enhance its bioavailability, thereby maximizing its potential therapeutic benefits (Figure 1). The materials obtained were characterized using Dynamic Light Scattering (DLS), and their antioxidant and cytotoxic activity was determined.

## 2. Results and Discussions

### 2.1. Esterification Between Arginine and Stearyl Alcohol

**Scheme 1:**
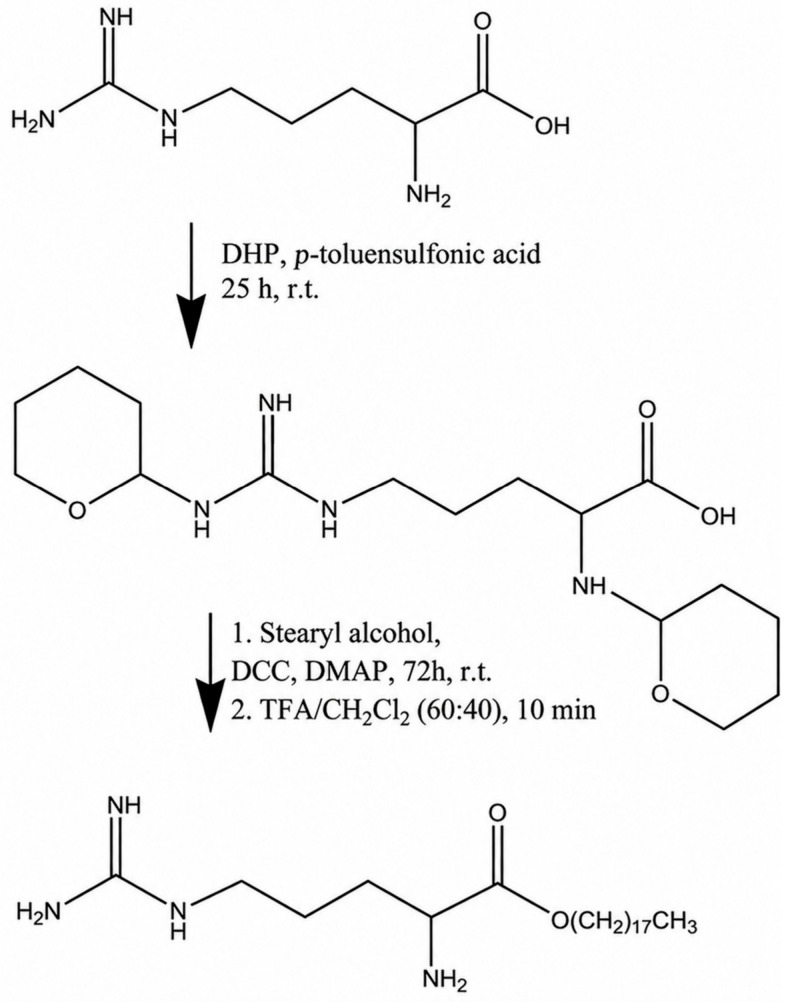
Synthetic route to arginine stearate.

The esterification reaction between arginine and stearyl alcohol was performed to obtain a sufficiently lipophilic compound to be used as a lipid matrix in SLNS for idebenone delivery, a synthetic analog of ubiquinone (also known as Coenzyme Q10). This synthesis was carried out in anhydrous dichloromethane (DCM) at room temperature (Scheme 1), after protecting the amino groups of arginine with THP.

The formation of protected arginine was confirmed by ^1^H-NMR. ^1^H-NMR (300 MHz, CD_3_OD): δ 1.50–1.95 (m, 16H, CH_2_ arginine and THP); 3.30–3.70 (m, 7H, CH_2_ and CH arginine, CH_2_O THP); 4.74 (m, 2H, CHNH THP). The formation of the ester was confirmed by FT-IR and ^1^H NMR. In particular, the FT-IR spectrum of the ester was compared with that of arginine and stearyl alcohol. The ester spectrum shows the presence of a new band at 1703 cm^−1^ which can be attributed to the stretching vibration of the C=O group of the ester. In addition, typical bands of NH stretching vibrations are observed at 3360 and 3180 cm^−1^ and those of CH2 groups at 2918 and 2875 cm^−1^. ^1^H-NMR (300 MHz, CD_3_OD): δ 0.88 (m, 3H, CH_3_); 1.26–1.43 (m, 30H, CH_2_); 1.51–1.88 (m, 6H, CH_2_ arginine and CH_2_ stearyl alcohol); 3.34–3.36 (m, 3H, CH_2_NH and CHNH_2_ arginine); 4.06 (m, CH_2_O stearyl alcohol.

### 2.2. Characterization of SLNSs

Empty SLNs and idebenone-loaded SLNs were successfully prepared using the microemulsion technique with high encapsulation efficiency, reaching 98%. This satisfactory result is likely due to the lipophilic characteristics of idebenone, which are well-suited to the lipophilic nature of the nanoparticles. Dynamic Light Scattering analysis allowed the determination of the average diameter of the nanoparticles and their polydispersity index (PI), as shown in Table 1. These PI values indicate good homogeneity in particle size distribution. While the chemical structure of the novel arginine stearate matrix features functional groups that could theoretically influence the nanoparticle surface properties, no direct experimental determination of the surface charge (such as Zeta potential) was performed in this study. Therefore, the discussion of the system’s performance is strictly confined to its hydrodynamic size and structural features.

### 2.3. TEM Analysis

**Figure 2:**
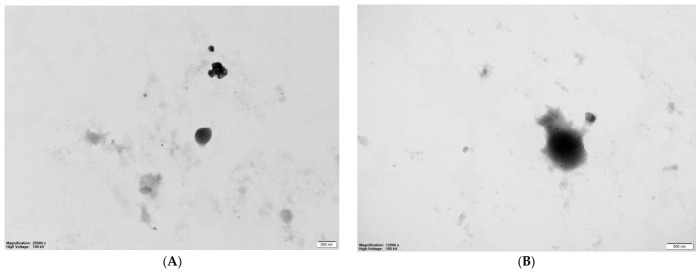
Representative transmission electron microscopy (TEM) micrographs of solid lipid nanoparticles (SLNs): (**A**) empty SLNs and (**B**) idebenone-loaded SLNs. Both formulations exhibited a predominantly spherical morphology with well-defined boundaries and a compact internal structure. Idebenone-loaded SLNs maintained the characteristic morphology of the empty formulation while showing a slightly larger size and increased electron density, consistent with successful drug incorporation within the lipid matrix. Occasional small aggregates were observed, likely resulting from solvent evaporation during TEM sample preparation. Scale bars: 200 nm (**A**) and 500 nm (**B**).

Transmission electron microscopy (TEM) analysis (Figure 2) demonstrated that both empty and idebenone-loaded solid lipid nanoparticles (SLNs) exhibited a predominantly spherical morphology with well-defined boundaries and a compact internal structure (Figure 2A,B). The empty SLNs (Figure 2A) appeared individually dispersed, with only occasional small aggregates, which are commonly attributed to solvent evaporation during TEM sample preparation. Following idebenone encapsulation (Figure 2B), the nanoparticles retained their characteristic spherical morphology, indicating that drug incorporation did not compromise the structural integrity of the lipid matrix. Compared with the empty formulation, the loaded SLNs appeared slightly larger and exhibited increased electron density, suggesting successful incorporation of idebenone within the lipid core. These observations are consistent with the dynamic light scattering (DLS) results, which showed an increase in the average hydrodynamic diameter from 287 nm for empty SLNs to 345 nm for idebenone-loaded SLNs. The discrepancy between TEM and DLS particle sizes is expected because TEM measures the dehydrated particle core, whereas DLS determines the hydrodynamic diameter of hydrated nanoparticles in suspension, including the surrounding solvation layer. Overall, the combined TEM and DLS analyses confirm successful nanoparticle formation and efficient idebenone encapsulation while preserving the characteristic spherical morphology of the system.

### 2.4. SLNs Characterization by DSC

**Figure 3:**
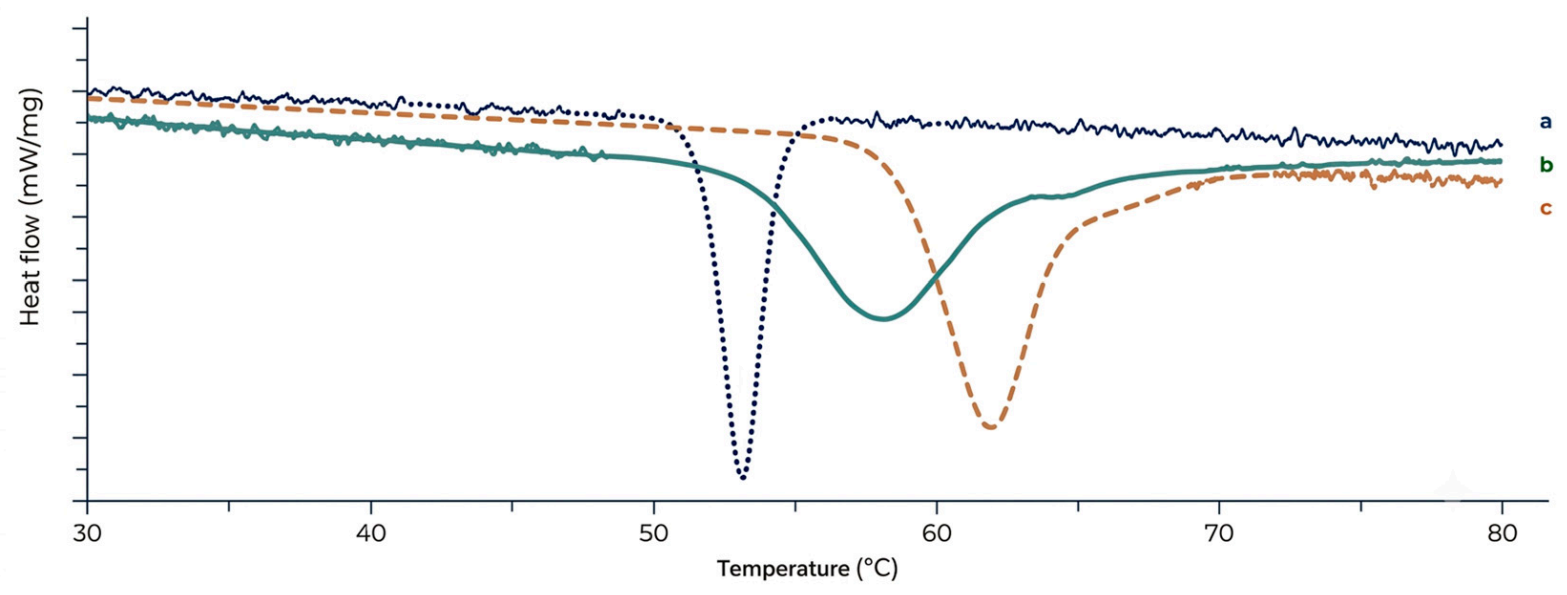
DSC analysis of Idebenone (**a**), Idebenone-loaded SLNs (**b**) and Arginine Stearate (**c**).

To clarify the physical state of idebenone within the lipid nanostructure, DSC analyses were carried out. The DSC thermogram of pure Idebenone (Figure 3a) showed a clear and intense endothermic melting peak at approximately 53.2 °C, indicative of its highly crystalline nature. In contrast, in the thermogram of the Idebenone-loaded SLNs (Figure 3b), this characteristic peak of the drug had completely disappeared. Instead, a single, broader endothermic peak was observed, corresponding to the lipid matrix, with a melting point slightly lower (~59.4 °C) than that of pure arginine stearate (~62.1 °C) Figure 3c, evidencing the thermal collapse of the ordered packing formed by the aliphatic chains of the stearate interacting with the hydrophilic heads of the amino acid. This significant shift towards a lower melting temperature (melting point depression) and the concomitant broadening of the peak can be scientifically explained by two concurrent factors: (i) the nanometric size of the lipid droplets, in which the high surface-to-volume ratio reduces the lattice energy required for melting according to the Gibbs-Thomson effect; (ii) the molecular dispersion of idebenone within the lipid core. The lipophilic drug acts as a ‘lattice defect’ or molecular impurity, disrupting the long-range crystalline order of the solid arginine stearate matrix.

### 2.5. In Vitro Release Studies

**Figure 4:**
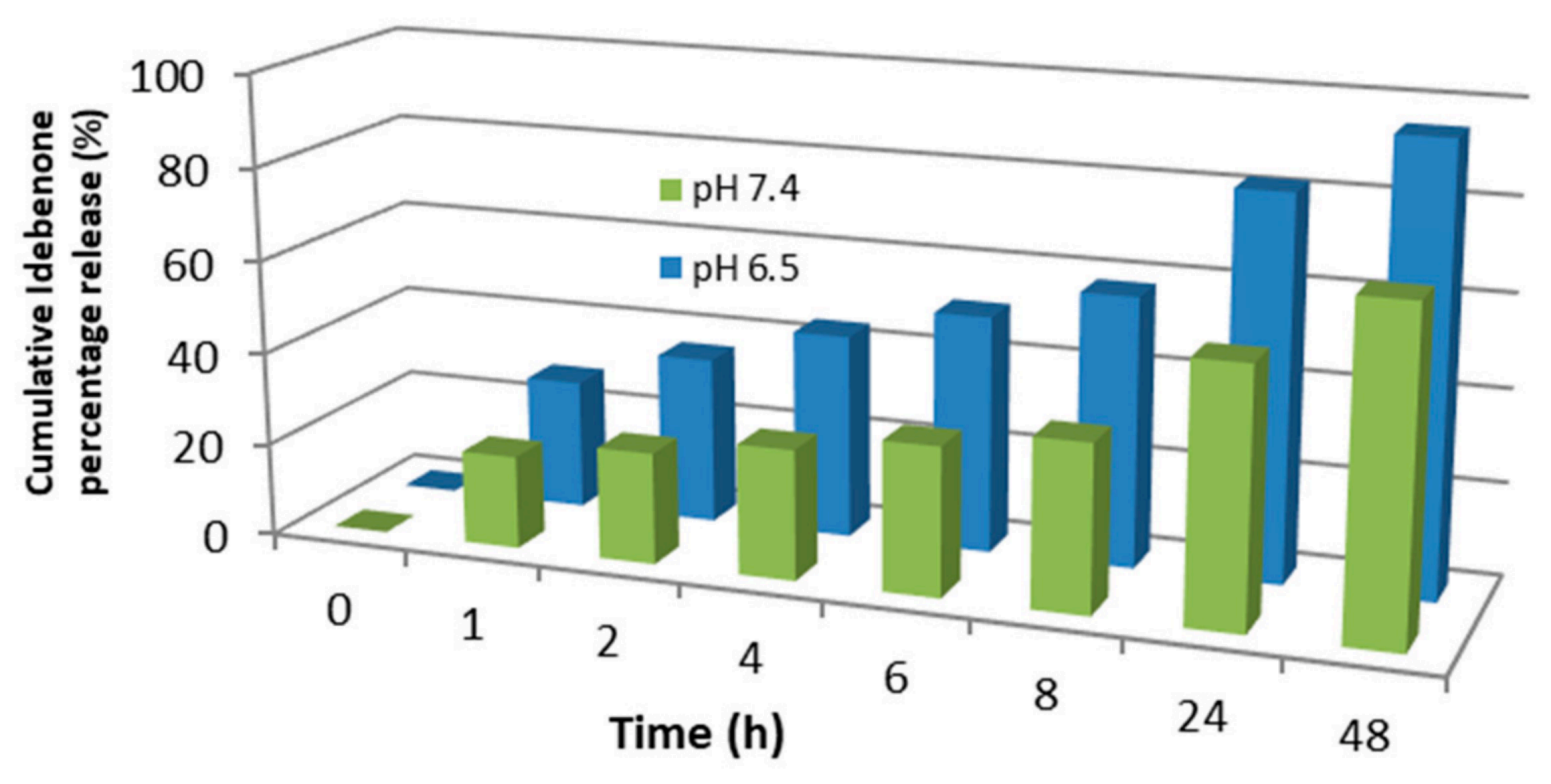
Release profile of idebenone assessed over a 48 h period at pH 7.4 and 6.5.

The in vitro release profile of idebenone from arginine stearate-based SLNs exhibited a biphasic pattern and differed according to the pH of the release medium. At pH 7.4, approximately 55% of the encapsulated idebenone was released after 24 h, whereas at pH 6.5, nearly 82% of the drug was released within the same time (Figure 4). These findings indicate that idebenone release was greater under mildly acidic conditions than at physiological pH. Although differences in the physicochemical properties of the lipid matrix under different pH conditions may contribute to the observed release behaviour, the present study was not designed to investigate the underlying release mechanism. Therefore, the observed pH-dependent release should be considered a comparative experimental observation rather than evidence of a specific mechanistic process. Further studies, including kinetic modelling and dedicated mechanistic investigations, will be necessary to clarify the factors governing idebenone release from these nanoparticles.

### 2.6. In Vitro Biological Activities

#### Antioxidant Activity Results

**Figure 5:**
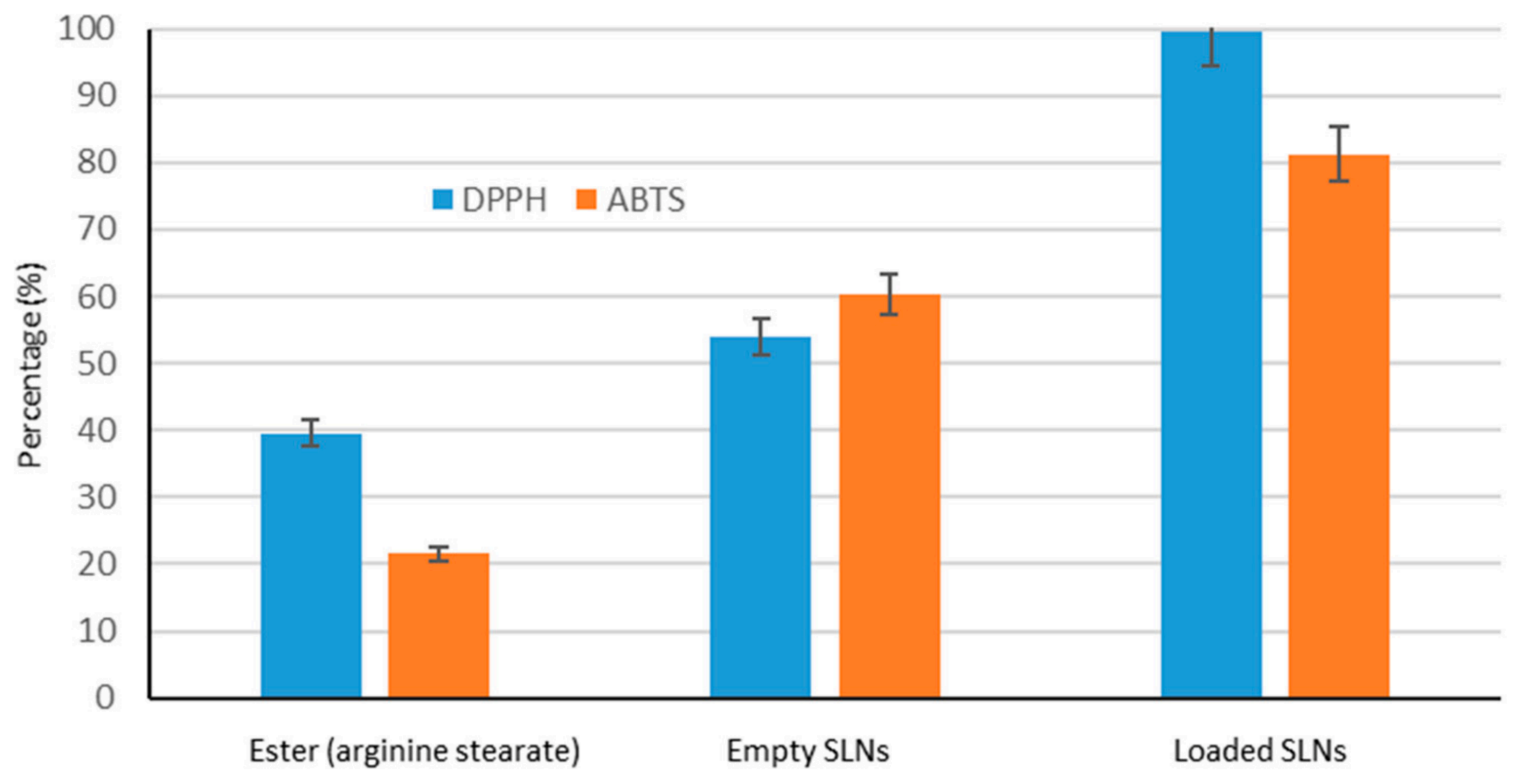
DPPH and ABTS antioxidant capacity of ester, empty and loaded SLNs. The DPPH and ABTS results are presented as mean ± SD.

Antioxidant activity was evaluated using the DPPH test and the ABTS test. Both tests were performed with the aim of evaluating radical-scavenging activity. The obtained results are expressed as a percentage, calculating the inhibition capacity using a standard formula that determines the antioxidant activity. This formula is based on absorbance readings obtained by spectrophotometry for the DPPH and ABTS assays. Regarding the DPPH assay, an initial absorbance reading of sample C was performed over a wavelength range of 400 to 900 nm, comparing it to the blank (“B” tube). Subsequently, an absorbance reading at 517 nm was performed for sample T, also compared to the blank, for a period of 10 min. The procedure was divided into two distinct cycles: in the first cycle, the absorbance reading was performed every 0.1 min for a period of 0.5 min. After the first reading, the sample was added. In the second cycle, readings were performed every 0.05 min for 9.5 min, thereby monitoring the evolution of absorbance over time and calculating the inhibition of the DPPH radical. The results of the DPPH assay showed a higher inhibition capacity for loaded SLNS, as reported in Figure 3. These values were obtained by calculating the change in absorbance and applying the DPPH inhibition formula. For the ABTS assay, the procedure was similar. After leaving the ABTS solution in the dark under a hood on a plate for 24 h, it was transferred to a cuvette for spectrophotometric analysis. The blank was prepared with 30 mL of ethanol and 1.5 mL of ABTS. Absorbance readings were taken at 734 nm, always compared to the blank, until the absorbance reached a value of 0.700. Once this reading was obtained, antioxidant activity was verified by mixing 10 mL of loaded SLNS with 3 mL of ABTS, 10 mL of empty SLNS with 3 mL of ABTS, and 10 mL of ester with 3 mL of ABTS. The purplish color that developed during the reaction indicated interaction with the ABTS radical, suggesting the presence of antioxidant activity in the samples (Figure 5).

In summary, the data obtained from the DPPH and ABTS assays allowed the calculation of the antioxidant activity of the different samples, confirming that the loaded and empty SLNS show high inhibition capacity, while the ester showed a more moderate antioxidant activity.

### 2.7. Anti-Glioblastoma Activity

**Figure 6:**
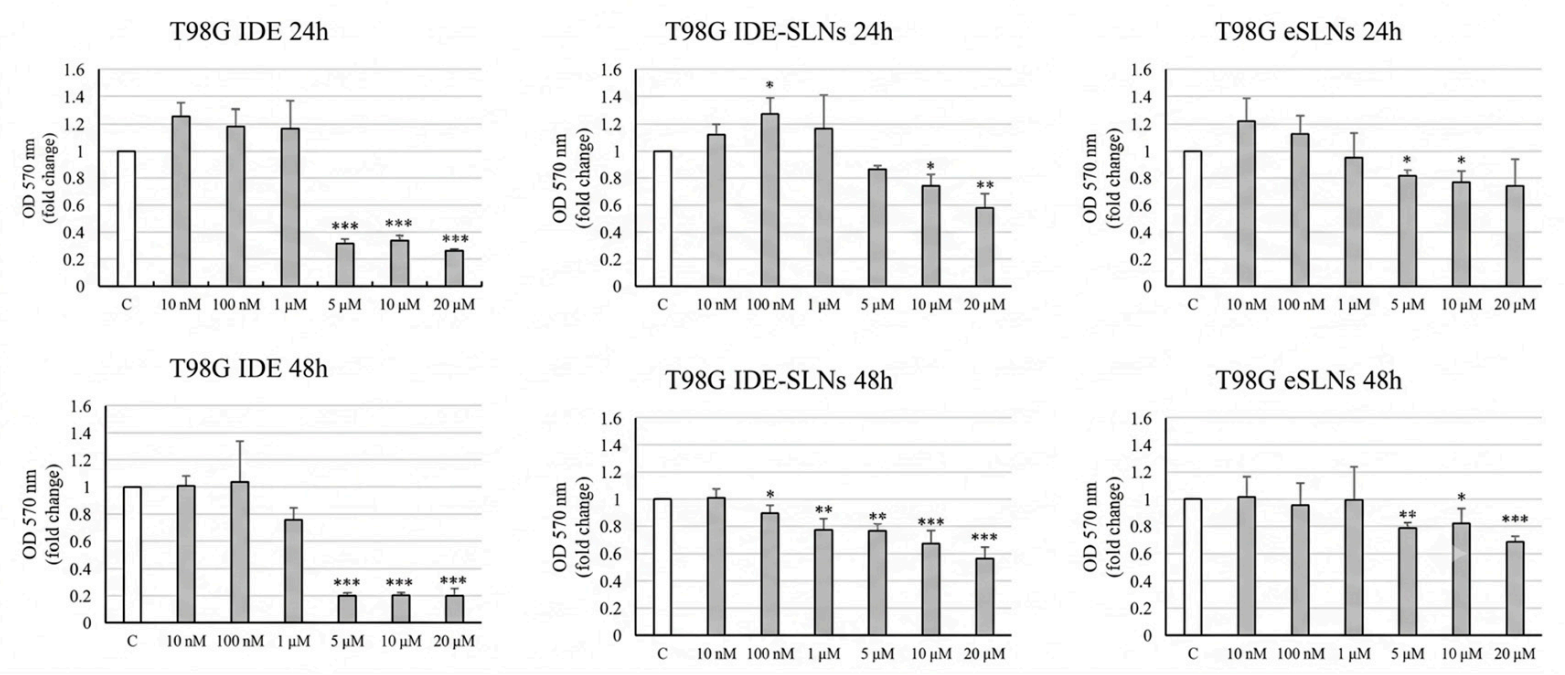
Effect of empty SNLs, SLNs-containing Idebenone and free Idebenone on cell survival in human glioblastoma cells, T98G, incubated for 24 and 48 h. The data represent the mean ± SEM of triplicates, Student T test was performed for statistical analysis: * *p* < 0.05, ** *p* < 0.01, *** *p* < 0.001.

To obtain a preliminary assessment of the biological activity of the developed formulation, T98G glioblastoma cells were selected as an in vitro model to evaluate the antiproliferative effect of IDE-SLNs. Cell viability was assessed by MTT assay following treatment of increasing concentrations of free idebenone, empty SLNs, or IDE-SLNs for 24 and 48 h. As shown in Figure 6, IDE-SLNs induced a concentration- and time-dependent reduction in T98G cell viability compared with control conditions. In contrast, empty SLNs exerted only a modest effect on cell viability, indicating that the observed antiproliferative activity was mainly attributable to idebenone. Free idebenone also reduced cell viability, particularly at the higher concentrations tested.

**Figure 7:**
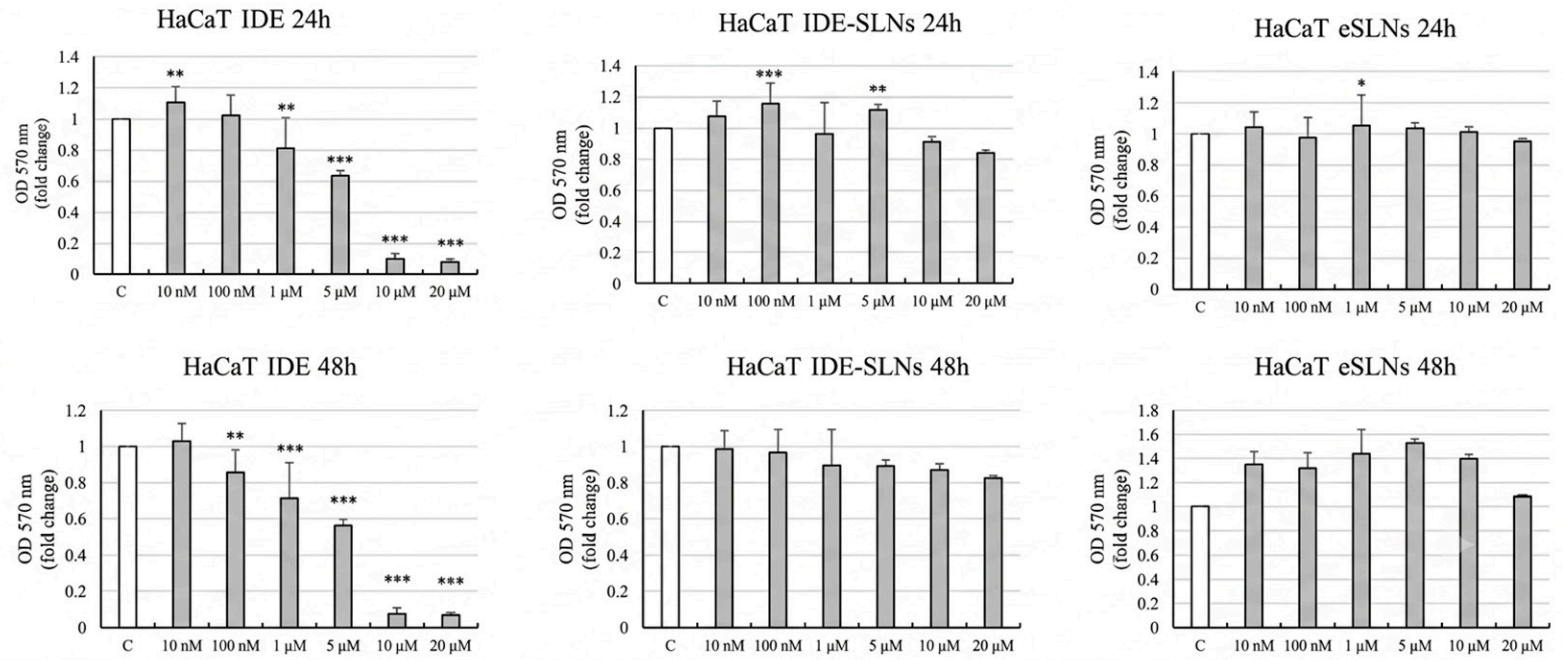
Impact of e SNLs, IDE-SLNs, and free IDE on the viability of human keratinocytes HaCaT cells under the same experimental conditions. Data are presented as mean ± SEM from triplicate experiments. Statistical significance was determined using the Student’s *t*-test: * *p* < 0.05, ** *p* < 0.01, *** *p* < 0.001.

To evaluate the preliminary cytocompatibility of the formulation toward non-tumor cells, HaCaT keratinocytes were treated under identical conditions. Neither empty SLNs nor IDE-SLNs significantly affected HaCaT cell viability at either 24 or 48 h, suggesting a favorable cytocompatibility profile of the developed formulation in this non-tumorigenic cell model (Figure 7). Conversely, free idebenone reduced HaCaT viability at the highest concentrations tested, suggesting that encapsulation into SLNs improves the cytocompatibility profile of idebenone toward non-tumorigenic cells.

### 2.8. Tability Assessment of SLNs

Both empty and idebenone-containing SLNS showed good stability over two months. However, during 2 months of storage at room temperature, an increase in particle size was observed, which became even more significant during storage at 37 °C. This behavior could be due to nanoparticle aggregation, which increases further with the introduction of thermal energy into the system.

## 3. Materials and Methods

### 3.1. Materials

The materials used for the synthesis processes are as follows: stearyl alcohol, 3,4-dihydro-2H-pyran (DHP), idebenone, dicyclohexylcarbodiimide (DCC), 4,4′-dimethylaminopyridine (DMAP), p-toluenesulfonic acid, trifluoroacetic acid (TFA), and arginine, purchased from Sigma Aldrich (St. Louis, MO, USA). The solvents used were diethyl ether, dichloromethane, ethanol, and hexane, purchased from Sigma Aldrich, VWR Chemicals (Radnor, PA, USA), Prolabo (Fontenay-sous-Bois, France), and Alfa Aesar (Ward Hill, MA, USA). For nanoparticle preparation, the following were used: Tween 20, 1-butanol, bile salt (sodium taurocholate hydrate), and distilled water, purchased from LabScan Analytical Sciences.

### 3.2. Instrumentation

**Figure 8:**
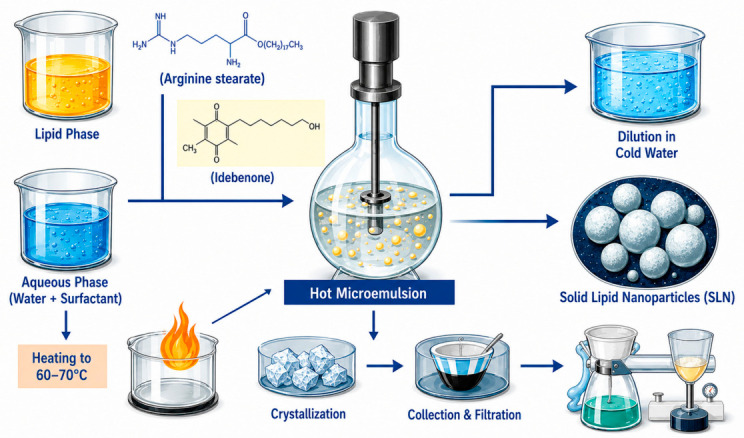
Preparation of solid lipid nanoparticles via microemulsion technique.

Nuclear Magnetic Resonance (^1^H-NMR) spectra were recorded at 300 MHz using a Bruker Avance 300 spectrometer. Deuterated dimethyl sulfoxide (DMSO-d_6_) was used as the solvent, and chemical shifts (δ) were reported in ppm relative to tetramethylsilane (TMS) as the internal reference. Reaction progress was monitored by thin-layer chromatography (TLC) using silica gel 60 F254 plates (Merck, Darmstadt, Germany), with visualization under UV light at 254 nm. Fourier-transform infrared (FT-IR) spectra were acquired using a Jasco 4200 spectrophotometer with potassium bromide (KBr) pellets prepared from KBr supplied by Sigma-Aldrich. UV–Vis spectra were recorded on a Jasco V-530 spectrophotometer (JASCO Corporation, Tokyo, Japan) using quartz cuvettes with a 1 cm optical path length. Nanoparticles were prepared using the microemulsification technique. Briefly, the lipid phase was melted on a hot plate, while the aqueous phase containing the surfactant was heated to the same temperature. Nanoparticle size distribution was determined using a Particle Size Analyzer 90 Plus (Brookhaven Instruments Corporation, New York, NY, USA). Solvents were removed under reduced pressure using a Büchi Rotavapor R II rotary evaporator (BÜCHI, Flawil, Switzerland), and compounds were freeze-dried using a Micro Modulyo lyophilization system (Edwards, Crawley, UK). The morphology of solid lipid nanoparticles (SLNs) was investigated by transmission electron microscopy (TEM). Figure 1 and Figure 8 were generated using Gemini AI based on prompts designed by the authors.

### 3.3. Arginine Protection

Arginine, DHP (in molar quantities double that of arginine), and p-toluenesulfonic acid (TsOH) (0.1 mol%) were introduced into a three-necked flask kept under an inert atmosphere and containing anhydrous dichloromethane, under magnetic stirring for 2 h, monitoring the reaction by TLC (thin-layer chromatography). Once the reaction was complete, the mixture was neutralized with aqueous sodium bicarbonate. The organic product was extracted and purified by column chromatography (hexane-dichloromethane 6:4). The obtained product was characterized by ^1^H-NMR.

### 3.4. Ester Synthesis

In a three-necked flask equipped with a reflux condenser, 0.776 g of protected arginine, 1.18 g of DCC, and 0.350 g of DMAP were introduced under magnetic stirring at room temperature with 150 mL of dichloromethane [20,21,22]. The reaction was left under stirring for 72 h. After 72 h, a white solution with a precipitate was evident, which, after chromatographic analysis on a silica gel plate, was filtered to obtain a solid component and a liquid organic component. The organic component was subjected to solvent removal by rotavapor, while the precipitate remained on the filter to be dried. Subsequently, the organic phase was pressurized using a diaphragm pump to eliminate any residual solvent and frozen, while the solid phase was washed with hot methanol to eliminate the dicyclohexylurea formed during the reaction, directly on the filter and dried using the same diaphragm pump. The liquid obtained from the second filtration was evaporated, subjected to acidic washing (TFA/CH_2_Cl_2_) for 10 min, and purified by column chromatography, using a hexane/chloroform 1:1 mixture. The obtained product was characterized by FT-IR and ^1^H-NMR.

### 3.5. Preparation of SLNSs

Solid lipid nanoparticles (SLNs) represent a promising platform for controlled drug release, thanks to their ability to improve the bioavailability of hydrophobic active ingredients and protect sensitive compounds from degradation. Among the various methods available for their preparation, the microemulsion technique is particularly appreciated for its simplicity and reproducibility [23,24]. This methodology initially involves the melting of the selected lipid, which is heated to a temperature above its melting point using a heating plate. In parallel, an aqueous phase containing the surfactant (and, in some cases, a co-surfactant) is heated to the same temperature as the lipid phase. It is crucial that both phases are brought to a uniform temperature to ensure proper microemulsion formation. Subsequently, the microemulsion is prepared by gradually adding the aqueous phase to the molten lipid phase under gentle stirring. This process facilitates the uniform dispersion of the lipid in the aqueous phase and the formation of a stable system characterized by low interfacial energy. The homogeneity of the system strongly depends on the selection of surfactants and their concentration, which must be optimized to minimize the risk of coalescence. Lipid nanoparticles are finally obtained by dispersing the hot microemulsion in cold water, maintained at a temperature between 2 °C and 10 °C. This critical step causes rapid cooling of the microemulsion, promoting lipid crystallization and the formation of solid nanoparticles. During this phase, the dispersion is mechanically stirred at a high speed, generally around 8000 rpm, for about 30 min. Mechanical stirring ensures a uniform distribution of nanoparticle sizes, reducing the possibility of aggregation (Figure 8). The microemulsion technique offers the advantage of not requiring the use of organic solvents, making it an environmentally friendly and safe method for pharmaceutical applications. However, to obtain optimal results, it is necessary to optimize parameters such as the lipid to surfactant ratio, the temperature of the phases, and the stirring speed, as these factors significantly influence the final properties of the SLNS, such as their size, stability, and drug loading capacity.

In one beaker, ester, idebenone (in the case of loaded SLN_S_), and a few drops of butanol were added, while in the second beaker, bile salt (hydrated taurodeoxycholate), butanol, H_2_O, and Tween 20 as an emulsifying agent were added. Table 2 shows the experimental conditions for preparing empty polymeric nanoparticles. The contents of the second beaker are poured into the first and heated to promote the complete dissolution of the lipid. Subsequently, the mixture is transferred into a three-necked flask containing 150 mL of H_2_O, leaving everything in an ice bath at low temperatures under magnetic stirring for about 30 min. At the end, the system is allowed to decant, and a few drops of the content from the surface are taken and placed in a cuvette with distilled H_2_O. This step is fundamental for an initial characterization of the SLNS, i.e., to determine their size through Light Scattering. The SLNS are filtered, frozen, and lyophilized to obtain a solid compound.

### 3.6. Nanoparticle Size Distribution Analysis

Nanoparticle size was determined by Dynamic Light Scattering (DLS) using a 90 Plus Particle Size Analyzer (Brookhaven Instruments Corporation, New York, NY, USA); the autocorrelation function was measured at 25 °C with a scattering angle of 90°. For size determination, quartz cuvettes were filled with 100 μL of sample solution and diluted with 4 mL of distilled water. In addition, the polydispersity index (PI), which indicates the degree of nanoparticle dispersion, was also determined. Three different measurements were performed to derive the average. The data were obtained using the “inverse Laplace transform” or Contin method. Stability studies were conducted on the SLNS samples to evaluate their size and polydispersity over time. Samples were stored in airtight jars, protected from light, at room temperature and at 37 °C for two months. Measurements of particle size and polydispersity index were performed at fixed time intervals (24 h, one week, two weeks, three weeks, one month, and two months) after preparation to monitor stability under the different storage conditions.

### 3.7. Characterization by Transmission Electron Microscopy (TEM)

Transmission electron microscopy was employed to characterize the morphology of CS-EVs. All of the TEM measurements were performed by depositing 20 μL of suspension of vesicles on a 300-mesh copper grid for electron microscopy covered by a thin amorphous carbon film. Samples were deposited at room temperature. Negative staining was realized by addition of10 μL of 2% aqueous phosphotungstic acid (PTA) solution (the pH was adjusted to 7.3 using 1 M NaOH). Measurements were carried out by using a FEI TECNAI 12 G2 Twin (FEI Company, Hillsboro, OR, USA), operating at 120 kV and equipped with an electron energy loss filter (Biofilter, Gatan Inc., Pleasanton, CA, USA) and a slow-scan charge-coupled device camera (794 IF, Gatan Inc., Pleasanton, CA, USA).

### 3.8. Idebenone Drug Content

To determine the encapsulation efficiency, the idebenone-loaded nanoparticles were separated from the unencapsulated (free) drug using centrifugation (Beckman Coulter, Brea, CA, USA) at 9500 rpm for 10 min. Following separation, the SLN breakdown occurred by sonication for 15 min at 40° in an 8:1 methanol/water mixture, promoting the release of the active ingredient. The amount of encapsulated idebenone was quantified via UV-Vis spectroscopy at 277 nm. Empty SLNS, after solvent evaporation, were placed in a quartz cuvette with the addition of distilled water. This sample served as a control. The same procedure was performed for SLNS loaded with the active ingredient, placed in a second cuvette. The encapsulation efficiency (EE%) was determined according to Equation (1):

(1) EE%=(gf/gi)×100

where gi represents the quantity of active substance used in the formulation of the SLNs, while gf indicates the quantity of active substance effectively encapsulated in the nanoparticles.

### 3.9. DSC Analysis

Calorimetric analyses of the samples were carried out using DSC (NETZSCH 200, NETZSCH-Gerätebau GmbH, Selb, Germany). Following a standard procedure, approximately 5.0 mg of dried sample was placed inside a sealed aluminum capsule and hermetically sealed with a sealed aluminum lid. Thermal analyses were carried out from 25 to 300 °C in a dry nitrogen atmosphere at a flow rate of 25 mL/min and a heating rate of 5 °C/min.

### 3.10. Evaluation of Antioxidant Activity

The antioxidant activity of the nanoparticles was evaluated through DPPH and ABTS assays, fundamental tools for determining the ability to eliminate free radicals. The DPPH (2,2-diphenyl-1-picrylhydrazyl) assay is a rapid, spectrophotometric method used to measure the antioxidant capacity of substances. It works by assessing the ability of antioxidants to reduce the stable, purple DPPH radical to yellow diphenylpicrylhydrazine, measured by a decrease in absorbance at 517 nm. The ABTS test (2,2-azino-bis(3-ethylbenzothiazoline-6-sulfonic acid)) is a spectrophotometric assay used to measure the antioxidant capacity of compounds. It works by measuring the ability of antioxidants to reduce the blue-green radical cation back to its colorless neutral form, typically monitored at 734 nm or 415 nm.

### 3.11. DPPH Assay

Samples containing 5 mg of ester and 1.5 mL of DPPH solution were prepared for both empty and charged nanoparticles. The samples were incubated for 30 min, during which a color change toward purple was observed. To ensure the stability of the molecule, the DPPH solution was stored in the refrigerator and protected from light with aluminum foil, as it is photosensitive. As a control, a sample composed of 50 mL of ethanol and 5 mg of DPPH was used. The antioxidant capacity was analyzed by UV-Vis spectrophotometry, monitoring the decrease in the absorption peak at 517 nm, characteristic of the DPPH radical. The antioxidant compounds present in the sample transfer hydrogen atoms to the radical, causing a discoloration proportional to their antioxidant charge. The DPPH radical scavenger activity was calculated according to the following Equation (2):

Scavenger Activity (%) = (A_0_ − A_1_)/A_0_ × 100 (2)

where A_0_ is the absorbance of the control (blank) and A_1_ is the absorbance in the presence of the SLNS. Results were expressed as mean ± SD.

### 3.12. ABTS Assay

For this assay, a solution was prepared by mixing 57.6 mg of ABTS in 15 mL of water with a second solution containing 3.3 mg of potassium persulfate in 30 mL of water. After mixing, the mixture turned green-blue and was left on a shaking plate in the dark for 24 h, protected from light by aluminum foil. The blank value obtained as a control was 0.748. In this test, the cationic radical ABTS^+^•, generated by the oxidation of the di-ammonium salt of 2,2′-azino-bis(3-ethylbenzothiazolino-6-sulfonic acid), has an absorption peak at 734 nm. Antioxidant compounds capable of donating a hydrogen atom or an electron to the cationic radical cause a discoloration proportional to their antioxidant activity. The antioxidant capacity was calculated using Equation (3):

(3) %Inhibition=(A0−As/A0)×100

where As is the absorbance of the sample at 734 nm and A_0_ is the control. Results were expressed as mean ± SD. The results of the DPPH and ABTS assays provided a quantification of the antioxidant power of the sample, highlighting its ability to neutralize free radicals.

### 3.13. Release Studies

Release studies were carried out on dialysis membranes (cutoff molecular weight 12–14 kDa) at different pH values (7.4 and 6.5), simulating the glioblastoma tumor microenvironment. Aliquots of empty nanoparticles and nanoparticles loaded with Idebenone (0.05 g) dissolved in various PBS solutions, depending on the pH under consideration, were placed in a beaker containing 25 mL of the same buffer solution. Finally, this beaker was placed in a thermostatic bath and stirred in the dark at 37 °C. At various time intervals (1 h, 2 h, 4 h, 6 h, 8 h, 24 h and 48 h), the solutions were analyzed spectrophotometrically and the percentage of drug released was expressed in relation to the absorbance.

### 3.14. Cell Culture Studies

#### 3.14.1. Cell Cultures

The human glioblastoma multiforme cell line, T98G, was purchased from ATCC (American Type Culture Collection). Cells were maintained in culture in MEM 1× medium (GIBCO) supplemented with 10% fetal bovine serum (FBS), 1% penicillin/streptomycin, 1% L-glutamine, 1% sodium pyruvate, and 1% non-essential amino acids. Human keratinocytes HaCaT were purchased from the American Type Culture Collection (ATCC, Manassas, VA, USA). The cells were stored in accordance with the supplier’s instructions and regularly tested to ensure they were free from mycoplasma (MycoAlert Mycoplasma Detection Assay, Lonza, Basel, Switzerland). HaCaT cells were maintained in culture in DMEM medium with 4500 mg/L glucose (Sigma Aldrich, Milan, Italy) supplemented with 10% FBS, 1% penicillin/streptomycin, and 1% L-glutamine. Both cell lines were cultured in a humidified incubator at 37 °C and 5% CO_2_.

#### 3.14.2. Cell Viability Assay

The MTT colorimetric assay measures the activity of the succinate dehydrogenase enzyme, present in cell mitochondria, which cleaves the tetrazolium ring of the yellow MTT molecule to produce insoluble purple formazan salts intracellularly. For this reason, MTT enters the cell and is correctly metabolized only if the cell is alive, leading to the formation of crystals that remain in the cell. Therefore, the amount of formazan produced is directly correlated with the number of living cells. To determine this, the crystals are analyzed by spectrophotometric reading of the sample at a wavelength of 570 nm. Both cell types were seeded in a 96-well multiwell plate at a confluence of 3000 cells/well and grown until complete adhesion. Before the start of the experiment, cells were starved in phenol red- and FBS-free medium for 24 h and then treated, in complete medium, with Idebenone (IDE), empty SLNS (eSLNs), and SLNS-containing idebenone (IDE-SLNs) at concentrations of 10 nM, 100 nM, 1µM, 5µM, and 10µM for 24 and 48 h. At the end of incubation, 100 μL of MTT (3-(4,5-dimethylthiazol-2)-2,5-diphenyltetrazolium bromide) at a concentration of 2 mg/mL was added to each well of the multiwell plate at 37 °C for 3 h. Subsequently, MTT was removed, and 100 μL of DMSO was added to each well to solubilize the formazan salts formed for 30 min. Finally, the absorbance values at 570 nm of the formazan solution in each well were evaluated using a 96-well multiwell spectrophotometer (MultiskanSkyHigh, Thermo Fisher Scientific, Life Technologies Holdings Pte. Ltd., Singapore).

### 3.15. Stability Studies

SLNS samples were stored in airtight jars and then kept in the dark at room temperature and at 37 °C for two months, separately. Particle size and polydispersity index of the samples were measured at fixed time intervals (24 h, one week, two weeks, three weeks, one month, and two months) after their preparation.

## 4. Conclusions

The primary objective of this work was the synthesis of a novel arginine stearate-based lipid matrix and its application in the formulation of solid lipid nanoparticles (SLNs) for the encapsulation of the antioxidant idebenone. The nanoparticles were successfully prepared using the microemulsion technique, achieving high encapsulation efficiency (98%), together with suitable particle size and a homogeneous size distribution. In vitro antioxidant assays (DPPH and ABTS) demonstrated that idebenone-loaded SLNs retained antioxidant activity, while the in vitro release study showed different release profiles at pH 7.4 and pH 6.5. These release data were intended to provide a comparative evaluation under different pH conditions and should not be interpreted as evidence of a specific release mechanism.

Preliminary biological evaluation in T98G glioblastoma cells and HaCaT keratinocytes showed that idebenone-loaded SLNs reduced the viability of T98G cells while maintaining good cytocompatibility toward non-tumorigenic HaCaT cells. These findings suggest that the developed formulation warrants further investigation as a lipid-based delivery system for idebenone.

Overall, this study demonstrates the successful development of arginine stearate-based SLNs as an efficient carrier for idebenone and provides a basis for further investigation. However, the present work does not include studies addressing blood–brain barrier permeability or brain delivery. Therefore, no conclusions regarding BBB penetration or enhanced cerebral delivery can be drawn from the current data. Future studies should include in vitro BBB models, transport and permeability assays, as well as in vivo investigations to evaluate the ability of these nanoparticles to reach the brain and to assess their therapeutic potential in glioblastoma and other neurological applications. In addition, further characterization of drug release kinetics, mechanistic studies, and evaluation in additional glioblastoma models will be necessary to better define the performance and potential applications of this formulation.

## Figures and Tables

**Table 1:** Average diameter and polydispersity index of SLNs.

Formulation	Average Particle Size (nm)	Polydispersity Index (PI)
Empty SLNs	286.9 ± 3.1	0.227 ± 0.013
Idebenone-loaded SLNs	345.0 ± 4.6	0.272 ± 0.047
Idebenone-loaded SLNs (2 months, 37 °C)	358.5 ± 5.2	0.281 ± 0.035

**Table 2:** Amount of reagent used in the preparation of SLNs.

SLNs	Lipid (G)	Tween20 (Ml)	Butanol (Ml)	Bile Salt (G)	H_2_O (Ml)	Idebenone (G)
Empty	0.05	0.016	0.0012	0.0036	0.059	-
Loaded	0.05	0.016	0.0012	0.0036	0.059	0.0053

## Data Availability

The data are contained within the article.
